# Ethnic Differences in Thrombotic Profiles of Acute Coronary Syndrome Patients and Relationship to Cardiovascular Outcomes: A Comparison of East Asian and White subjects

**DOI:** 10.1055/s-0043-1777794

**Published:** 2023-12-29

**Authors:** Jung-Won Suh, Vassilios Memtsas, Ying X Gue, Hyoung-Won Cho, Wonjae Lee, Si-Hyuck Kang, Diana A. Gorog

**Affiliations:** 1 Department of Internal Medicine, Seoul National University Bundang Hospital, Seoul National University College of Medicine, Seongnam, Korea; 2 Cardiovascular Division, Faculty of Medicine, National Heart and Lung Institute, Imperial College, London, United Kingdom; 3 Liverpool Centre for Cardiovascular Science, University of Liverpool and Liverpool Heart & Chest Hospital, Liverpool, United Kingdom; 4 Centre for Health Services and Clinical Research, Postgraduate Medical School, University of Hertfordshire, Hertfordshire, United Kingdom

**Keywords:** myocardial infarction, ethnicity, thrombotic status, fibrinolysis, East Asian

## Abstract

**Background** East Asians (EAs), compared to white Caucasians (W), have a lower risk of ischemic heart disease and a higher risk of bleeding with antithrombotic medications. The underlying mechanisms are incompletely understood.

**Objectives** We sought to compare thrombotic profiles of EA and W patients with myocardial infarction (MI) and relate these to cardiovascular outcomes.

**Methods** In a prospective study in the United Kingdom and Korea, blood samples from patients ( *n* = 515) with ST- or non-ST-elevation MI (STEMI and NSTEMI) were assessed using the Global Thrombosis Test, measuring thrombotic occlusion (OT) and endogenous fibrinolysis (lysis time [LT]). Patients were followed for 1 year for major adverse cardiovascular events (MACE) and bleeding.

**Results** EA patients showed reduced OT (longer OT) compared to W (646 seconds [470–818] vs. 436 seconds [320–580], *p* < 0.001), with similar LT. In STEMI, OT (588 seconds [440–759] vs. 361 seconds [274–462], *p* < 0.001) and LT (1,854 seconds [1,389–2,729] vs. 1,338 seconds [1,104–1,788], *p* < 0.001) were longer in EA than W. In NSTEMI, OT was longer (OT: 734 seconds [541–866] vs. 580 seconds [474–712], *p* < 0.001) and LT shorter (1519 seconds [1,058–2,508] vs. 1,898 seconds [1,614–2,806], *p* = 0.004) in EA than W patients. MACE was more frequent in W than EA (6.3 vs. 1.9%, *p* = 0.014) and bleeding infrequent. While OT was unrelated, LT was a strong independent predictor of MACE event after adjustment for risk factors (hazard ratio: 3.70, 95% confidence interval: 1.43–9.57, *p* = 0.007), predominantly in W patients, and more so in STEMI than NSTEMI patients.

**Conclusion** EA patients exhibit different global thrombotic profiles to W, associated with a lower rate of cardiovascular events.

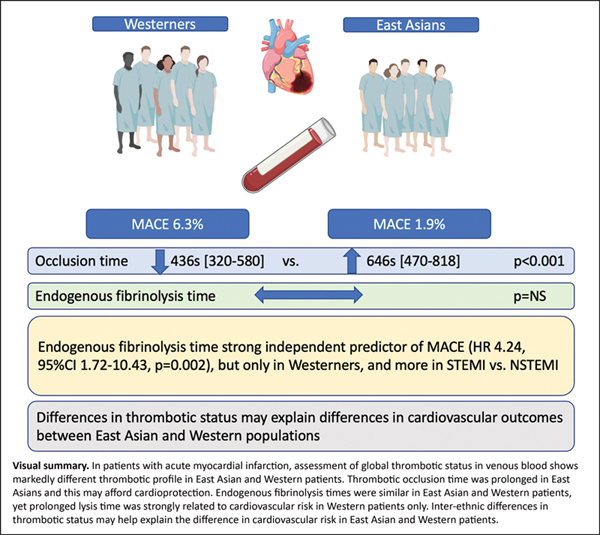

## Introduction

Multiple lines of evidence indicate that East Asian individuals have a lower risk of atherothrombotic coronary events compared to Caucasians, and a relatively higher risk of major bleeding complications, including intracranial hemorrhage with antithrombotic medications. 1 2 3 Annualized standardized death rates from ischemic heart disease are the lowest in Japan (32.97 [31.82–34.07]) and South Korea (34.03 [29.65–34.51]), at <30% of the average global level. 4 A study comparing the posthospital outcomes of acute myocardial infarction (AMI) in Japanese and North Americans revealed a significantly greater risk of adverse cardiovascular events in North American patients. 5

The mechanisms that underlie this phenomenon are incompletely understood. Clinical characteristics of East Asians differ from those of Westerners, generally manifesting lower body mass index (BMI) and total cholesterol, with more frequent hypertension. 3 There is likely to be a lower genetic predisposition to coronary disease in East Asians, with interaction between genetic and epigenetic factors contributing to inter-ethnic disparity. The Framingham risk score markedly overestimates the absolute cardiovascular risk in East Asians. 6 7 8 An international registry of 16,451 patients evaluating the prognostic significance of coronary disease on computed tomographic angiography showed the annualized incidence of death or AMI with obstructive coronary disease (≥50% stenosis) was 2.2% in Caucasians and 0.8% in East Asians, and compared to other ethnicities, East Asians had fewer events than expected (adjusted hazard ratio [HR]: 0.25; 95% confidence interval [CI]: 0.16–0.38; *p* < 0.001). 9

Genetic differences may also underlie lower coagulant and inflammation levels in East Asians compared to Caucasians. There is a close relationship between high-sensitivity C-reactive protein (hs-CRP) level and cardiovascular events, 10 11 12 and among different ethnicities, East Asians have the lowest levels of inflammation. 13 14 15 A large Korean registry of 268,803 subjects in a screening program showed low hs-CRP levels and a significant linear association with cardiovascular disease. 11 Endothelial activation markers such as ICAM-1, E-selectin, and von Willebrand factor and coagulation markers including plasminogen activator inhibitor 1, fibrinogen, thrombin, and factor VII and VIII levels tend to be lower in East Asian than in Caucasian patients. 16 17 18 The “East Asian Paradox” refers to the finding of an enhanced pharmacokinetic and pharmacodynamic effect with most antithrombotic medications in East Asians compared to Caucasians, including P2Y _12_ inhibitors and oral anticoagulants, resulting in more frequent bleeding and consequent recommendations for reduced dose antithrombotic prescribing regimens in East Asian patients. 19 20 21 22

Whilst individual markers of coagulation and fibrinolysis have variably been linked to atherothrombotic risk, the overall thrombotic profile is difficult to ascertain from individual biomarkers, such that global tests of thrombotic status are preferable. 23 24 25 Recently, impaired endogenous fibrinolysis has emerged as a novel, independent risk factor for recurrent cardiovascular events in patients with AMI. 26 27

Whether thrombotic profiles of East Asian and Western patients with AMI differ and whether this relates to cardiovascular outcomes are unknown. We sought to compare thrombotic profiles of East Asian and Western patients with AMI and relate these to cardiovascular outcomes.

## Methods

### Study Design

We conducted a prospective observational cohort study in two centers, namely Seoul National University Bundang Hospital (SNUBH), Seongnam, South Korea and The Lister Hospital, East and North Hertfordshire NHS Trust, Hertfordshire, United Kingdom. The studies were performed in accordance with the Declaration of Helsinki and Good Clinical Practice and their design was approved by the institutional review board of SNUBH (B-209-634-303) and the UK National Health Research Authority (IRAS 26078, REC 19/LO/0390) (Clinicaltrials.gov identifier: NCT02562690, IRAS ID: 260786). Patients were enrolled between in the United Kingdom between February 18, 2016 and May 30, 2021. In Korea, the participants were enrolled between April 20, 2017 and May 30, 2021.

### Study Population

Consecutive eligible patients of corresponding ethnicity presenting with ST-segment elevation or non-ST segment elevation MI (STEMI and NSTEMI) were recruited in the United Kingdom and South Korean centers, respectively. Adults (≥18 years) with a presumed diagnosis of STEMI or NSTEMI based on clinical presentation, electrocardiogram, and biochemical criteria were enrolled. 28 29 Patients receiving oral anticoagulation, those with known coagulation disorder, sepsis, platelet count <100 × 10 ^9^ /L, hemoglobin <80 g/L, active malignancy, those unable to take dual antiplatelet therapy (DAPT), or already enrolled in an interventional research trial were excluded.

All patients gave written informed consent. A delayed consent strategy was used, with ethical approval, in patients presenting with STEMI. In these patients, upon arrival to the hospital, in addition to routine blood tests, an extra blood sample was obtained to assess baseline thrombotic status through the same blood draw. Patients then underwent emergency angiography and primary percutaneous coronary intervention (pPCI) as clinically indicated, as well as standard-of-care medications including guideline-directed DAPT as determined by the treating clinician and institutional protocols. Surviving STEMI patients were subsequently approached for consent. Patients who died before consent could be obtained were excluded. Patients presenting with NSTEMI were approached on the day or the day after admission to seek consent. Patients received standard-of-care medications including guideline-directed DAPT and underwent percutaneous coronary intervention as determined by the treating clinician and institutional protocols. In NSTEMI patients, blood samples for thrombotic status were obtained only after discontinuation of anticoagulant medication (subcutaneous factor Xa inhibitor or low-molecular-weight heparin) for at least 48 hours. This meant that the majority of NSTEMI patients were sampled 48 to 96 hours after admission (and therefore >24 hours after DAPT loading).

Antiplatelet therapy on admission consisted of a “loading” dose of aspirin 300 mg and either clopidogrel 600 mg or ticagrelor 180 mg or prasugrel 60 mg, given in the ambulance or emergency department upon diagnosis. For the UK cohort, patients given clopidogrel by the ambulance crew received additional ticagrelor 180 mg loading dose peri-pPCI and continued this postprocedure. In the vast majority of patients with STEMI, DAPT loading occurred in the 30 minutes preceding blood sampling on arrival. Unfractionated heparin 70 to 100 IU/kg was given immediately pre-pPCI. Use of glycoprotein IIb/IIIa inhibitor or bivalirudin (Angiomax, The Medicines Company, Parsippany, New Jersey, United States), decisions regarding access site, thrombus aspiration, and stent type were left to the treating physician.

### Blood Sampling

In STEMI patients, nonfasting blood samples were taken upon arrival, after DAPT loading, prior to heparin administration, and before pPCI. Samples were taken from a 6-Fr radial or femoral sheath, which was flushed with nonheparinized saline before insertion. A two-syringe technique was employed, using the first 5 mL for routine tests and the second 5 mL for assessment of thrombotic status. For NSTEMI patients, nonfasting blood samples were taken either from the 6-Fr arterial sheath or from a large bore, usually antecubital, vein, using an 18-G butterfly cannula. A two-syringe technique was employed, using the first 5 mL for routine tests and the second 5 mL for thrombotic status assessment, avoiding prolonged tourniquet time. Based on previous internal validation data performed by our group, no difference in thrombotic status between simultaneously collected arterial and venous sampling routes was observed.

### Assessment of Thrombotic Status

Venous blood samples were tested upon withdrawal with the point-of-care Global Thrombosis Test (GTT) (Thromboquest Ltd, London, United Kingdom). This automated technique utilizes nonanticoagulated native whole blood to determine global thrombotic and fibrinolytic status. The instrument was positioned in the catheterization laboratory, ready to use, for patients presenting with STEMI. For patients presenting with NSTEMI, blood samples were obtained during hospitalization on the ward with the GTT instrument positioned near the patient. The native blood sample taken from the patient was immediately introduced into the GTT cartridge in the instrument within 15 seconds (s) of withdrawal and the automated measurement begun. In the cartridge, blood flows through a conical plastic tube, passing through small gaps adjacent to two sequential beads. As blood flows through the gaps adjacent to the upper bead, the resulting initial high shear stress (180 dynes/cm ^2^ ) causes platelet activation. Immediately downstream, in the low shear zone between the beads, the activated platelets aggregate, thrombin is generated, and eventually the growing micro-thrombi occlude the gaps adjacent to the second bead, reducing the flow rate and finally arresting flow. The instrument measures the time (d) between consecutive blood drops at the exit of the conical part of the tube, which gradually increases as thrombi start to occlude the gaps adjacent to the second bead and at an arbitrary point (d ≥ 15 s), the instrument records, and displays occlusion time (OT; seconds). The restart of blood flow following occlusion is due to spontaneous thrombolysis (lysis time, LT; seconds). If lysis does not occur until 6,000 seconds following OT (LT cut-off time), “no lysis” is recorded. The principle of the technique, as well as inter- and intra-assay coefficients of variation, has been previously described. 27 30 Both centers used factory-calibrated instruments obtained from the same manufacturer. Finally, investigators in both sites utilized the same sample handling and near patient testing technique as described above, to minimize variability between the two centers.

### Data Collection and Follow-Up

During the index admission, case notes and electronic records were examined, to allow contemporaneous completion of study-specific case record forms. Patients were followed-up for 12 months for the occurrence of study endpoints.

### Study Endpoints

The primary endpoint was the occurrence of major adverse cardiovascular events (MACE), defined as the composite of cardiovascular death, nonfatal MI including stent thrombosis (defined according to the Academic Research Consortium criteria), and stroke/transient ischemic attack (definitions in Supplementary Material , available in the online version). Secondary endpoints included the individual components of the primary endpoint and the occurrence of major bleeding (grades 3–5) classified according to the Bleeding Academic Research Consortium definition, as well as all-cause death and repeat revascularization. 31 For all endpoints, hospital and primary care (where available) source documents were obtained, and diagnosis verified by two independent clinicians blinded to thrombotic status results in both centers.

### Statistical Analysis

The study aimed to assess whether LT is a predictor of MACE in patients with AMI and evaluate the racial disparity of adverse events between the two ethnic groups (Westerners and East Asians). A recent study comparing similar thrombotic and thrombolytic activity in patients with AMI reported LT values of 1,664 ± 756 seconds. 32 Using the independent *t* -test and assuming a one-sided alpha of 0.05, 5% MACE, and 10% attrition rate, we calculated that 493 patients would be required to achieve a power 1-β >0.80, to determine a 12% difference in LT for an enrolment ratio of 1:1 between the two groups.

Data are presented as mean (standard deviation) or median (interquartile range). Dichotomous variables were compared using Fisher's exact test. Correlations were analyzed using Spearman's method. The ability of the test to discriminate between patients with and without the study endpoint was evaluated by receiver-operating characteristic (ROC) curve analysis. Only events occurring beyond the point of LT testing were related to the test's ability to predict MACE. To investigate the relationship between LT and MACE, univariate and multivariable hazard regression models of Cox were used. Study variables were first analyzed with univariate analysis and those risk factor variables that showed a significant interaction ( *p* < 0.05) were entered into the multivariable analysis. Analyses were performed with Stata V.17 (StataCorp, College Station, Texas, United States).

## Results

We recruited 255 patients from the United Kingdom and 260 from South Korea. The clinical characteristics and baseline laboratory characteristics of all patients on admission, and medications on discharge, are shown in Table 1 , and subgroups of STEMI and NSTEMI patients shown in Tables 2 and 3 . The groups were generally well matched, with notable exceptions for raised BMI, hypercholesterolemia, peripheral arterial disease, and family history of cardiovascular disease, all more prevalent in the Western patients. Potent P2Y _12_ inhibitors prasugrel or ticagrelor were more frequently prescribed for Western patients than South Korean patients upon discharge (74 vs. 38%, *p* < 0.001).

**Table 1:** Baseline clinical and laboratory characteristics of whole study cohort

	Western patients ( *n* = 255)	East Asian patients ( *n* = 260)	*p* -Value
*Clinical characteristics*			
Age, y (SD)	66.3 (12.4)	64.5 (12.4)	0.140
Male, *n* (%)	197 (77.3)	210 (80.8)	0.384
Ethnicity			
• White	255	0	
• East Asian	0	260	
• Black	0	0	
• Asian	0	0	
• Hispanic	0	0	
BMI, kg/m ^2^ (SD)	27.7 (5.2)	24.7 (3.7)	**<0.001**
Smoking, *n* (%)	61 (23.9)	71 (27.3)	0.436
Hypertension, *n* (%)	132 (51.8)	134 (51.5)	1.000
Diabetes, *n* (%)	59 (23.1)	71 (27.3)	0.323
Hypercholesterolemia, *n* (%)	85 (33.3)	54 (20.8)	**0.002**
Family history of premature CAD, *n* (%)	81 (31.8)	61 (23.5)	**0.044**
Angina, *n* (%)	23 (9.0)	35 (13.5)	0.145
Prior MI, *n* (%)	32 (12.6)	35 (13.5)	0.860
Prior PCI, *n* (%)	30 (11.8)	37 (14.2)	0.484
Prior CABG, *n* (%)	4 (1.6)	0 (0)	0.119
CKD, *n* (%)	11 (4.3)	8 (3.1)	0.610
PAD, *n* (%)	8 (3.1)	1 (0.4)	**0.035**
CVA, *n* (%)	8 (3.1)	11 (4.2)	0.673
*ACS presentation*			
STEMI, *n* (%)	160 (62.8)	160 (61.5)	0.786
NSTEMI, *n* (%)	95 (37.2)	100 (38.5)	0.786
*Laboratory characteristics*			
Hb, g/dL (IQR)	14.2 (13.2–15.2)	13.8 (12.7–14.9)	**0.005**
WCC, ×10 ^9^ /L (IQR)	9.8 (8.0–12.1)	9.3 (7.2–11.7)	**0.035**
Platelets, ×10 ^9^ /L (IQR)	248 (202–291)	221 (184–255)	**<0.001**
INR, (IQR)	1 (0.9–1)	1 (0.9–1)	**0.026**
PT, s (IQR)	11.2 (10.7–11.8)	12.8 (12.4–13.5)	**<0.001**
APTT, s (IQR)	26.2 (23.6–29.3)	34.5 (31.6–37.9)	**<0.001**
Fibrinogen, g/L (IQR)	4.1 (3.4–5)	3.4 (2.9–3.9)	**<0.001**
Creatinine, μmol/L (IQR)	84 (72–102)	82 (70–97)	0.191
hs-CRP, mg/L (IQR)	3 (1–7)	4 (3–10.7)	**<0.001**
HbA1c, mmol/mol (IQR)	44 (37–59)	42.5 (38–52)	0.746
Glucose, mmol/L (IQR)	7.6 (6–11.5)	7.7 (6.4–9.9)	0.802
Total cholesterol, mmol/L (IQR)	5.1 (4.1–5.8)	4.2 (3.4–5.1)	**0.000**
LDL cholesterol, mmol/L (IQR)	2.5 (1.5–3.4)	2.6 (1.9–3.4)	0.287
Troponin (peak)			
hs-troponin T, ng/L (IQR)	735 (93–2463)	n/a	
hs-troponin I, ng/L (IQR)	n/a	34,873 (5,787–86,584)	
*Thrombotic status*			
OT, s (IQR)	436 (320–580)	646 (470–818)	**<0.001**
LT, s (IQR)	1,542 (1,171–2,160)	1,735 (1,238–2,669)	0.052
*Admission medications* a			
Aspirin, *n* (%)	58 (22.8)	114 (43.9)	**<0.001**
*Discharge medications*			
Aspirin, *n* (%)	240 (94.1)	246 (94.6)	0.139
Clopidogrel, *n* (%)	47 (18.4)	158 (60.8)	**<0.001**
Ticagrelor, *n* (%)	188 (73.7)	51 (19.6)	**<0.001**
Prasugrel, *n* (%)	0 (0.0)	46 (17.7)	**<0.001**
Beta-blocker, *n* (%)	218 (85.5)	200 (76.9)	**0.001**
ACEi/ARB, *n* (%)	223 (87.5)	199 (76.5)	**<0.001**
Statin, *n* (%)	232 (90.9)	243 (93.5)	0.834

Abbreviations: ACEi, angiotensin-converting enzyme inhibitor; ACS, acute coronary syndrome; APTT, activated partial thromboplastin time; ARB, angiotensin receptor blocker; BMI, body mass index; CABG, coronary artery bypass grafting; CAD, coronary artery disease; CKD, chronic kidney disease; CVA, cerebrovascular accident; Hb, hemoglobin; HbA1c, hemoglobin A1c; hs-CRP, high-sensitivity c-reactive protein; hs-troponin I, high-sensitivity troponin I; hs-troponin T, high-sensitivity troponin T; INR, international normalized ratio; IQR, interquartile range; LDL, low-density lipoprotein; LT, lysis time; MI, myocardial infarction; NSTEMI, non-ST segment elevation myocardial infarction; OT, occlusion time; PAD, peripheral arterial disease; PCI, percutaneous coronary intervention; PT, prothrombin time; SD, standard deviation; STEMI, ST-segment elevation myocardial infarction; WCC, white cell count.

Note: Values are presented as mean (SD), median (IQR), or *n* (%). CKD defined as creatinine >177 μmol/L. Prior aspirin defined as regular use prehospitalization. Family history of premature CAD defined as a diagnosis of CAD in a first-degree relative <60 years. Values in bold are significant (i.e., *p* < 0.05).

a Refers to relevant medications that the patients were taking up to the time of admission, i.e., on a regular basis at home. None were taking a P2Y _12_ inhibitor at prior to admission.

**Table 2:** Baseline clinical and laboratory characteristics of patients with STEMI

	Western patients ( *n* = 160)	East Asian patients ( *n* = 160)	*p* -Value
*Clinical characteristics*			
Age, y (SD)	65.8 (12.2)	61.4 (12.3)	**0.002**
Male, *n* (%)	128 (80)	134 (83.8)	0.385
BMI, kg/m ^2^ (SD)	27.3 (4.7)	24.6 (3.8)	**<0.001**
Smoking, *n* (%)	42 (26.3)	53 (33.1)	0.179
Hypertension, *n* (%)	82 (51.2)	73 (45.6)	0.315
Diabetes, *n* (%)	32 (20.0)	33 (20.6)	0.890
Hypercholesterolemia, *n* (%)	60 (37.5)	35 (21.9)	**0.003**
Family history of premature CAD, *n* (%)	55 (34.4)	42 (26.3)	0.114
Angina, *n* (%)	12 (7.5)	18 (11.3)	0.251
Prior MI, *n* (%)	15 (9.4)	18 (11.3)	0.582
Prior PCI, *n* (%)	14 (8.8)	21 (13.1)	0.211
Prior CABG, *n* (%)	1 (0.6)	0 (0.0)	1.000
CKD, *n* (%)	7 (4.4)	3 (1.9)	0.199
PAD, *n* (%)	6 (3.8)	1 (0.6)	0.121
CVA, *n* (%)	5 (3.1)	5 (3.1)	1.000
*Laboratory characteristics*			
Hb, g/dL (IQR)	14.1 (13.0–15.2)	14.2 (12.9–15.1)	0.830
WCC, ×10 ^9^ /L (IQR)	10.5 (8.6–12.4)	10.4 (8.3–13.0)	0.825
Platelets, ×10 ^9^ /L (IQR)	241 (200–282)	226 (188–263)	**0.012**
INR (IQR)	1 (0.9–1.0)	1.0 (0.9–1.0)	0.660
PT, s (IQR)	11.5 (10.9–12.1)	12.8 (12.4–13.4)	**<0.001**
APTT, s (IQR)	28.6 (26.3–30.6)	33.7 (31.2–37.9)	**<0.001**
Fibrinogen, g/L (IQR)	4.6 (3.8–5.3)	3.3 (2.8–3.9)	**<0.001**
Creatinine, μmol/L (IQR)	84 (72–101)	82 (69–95)	0.092
hs-CRP, mg/L (IQR)	3 (1–8)	4 (3–15)	**0.002**
HbA1c, mmol/mol (IQR)	52 (48–60)	42 (38–52)	**0.028**
Glucose, mmol/L (IQR)	11.7 (9.6–12.9)	8.7 (6.9–11.0)	**0.001**
Total cholesterol, mmol/L (IQR)	5.1 (4.3–5.9)	4.5 (3.7–5.3)	**<0.001**
LDL cholesterol, mmol/L (IQR)	2.6 (1.9–3.8)	2.8 (2.0–3.5)	0.474
Troponin (peak)			
hs-troponin T, ng/L (IQR)	1,867 (838–3640)	n/a	
hs-troponin I, ng/L (IQR)	n/a	68,416 (24,783–109,984)	
*Thrombotic status*			
OT, s (IQR)	361 (274–462)	588 (440–759)	**<0.001**
LT, s (IQR)	1,338 (1,104–1,788)	1,854 (1,389–2,729)	**<0.001**
*Admission medications*			
Aspirin, *n* (%)	30 (18.8)	41 (25.6)	0.178
*Discharge medications*			
Aspirin, *n* (%)	148 (92.5)	153 (95.6)	0.385
Clopidogrel, *n* (%)	25 (15.6)	78 (48.8)	**<0.001**
Ticagrelor, *n* (%)	122 (76.3)	44 (27.5)	**<0.001**
Prasugrel, *n* (%)	0 (0.0)	33 (20.6)	**<0.001**
Beta-blocker, *n* (%)	139 (86.9)	135 (84.4)	0.054
ACEi/ARB, *n* (%)	143 (89.4)	133 (83.1)	**0.001**
Statin, *n* (%)	147 (91.9)	154 (96.3)	0.823

Abbreviations: ACEi, angiotensin-converting enzyme inhibitor; ACS, acute coronary syndrome; APTT, activated partial thromboplastin time; ARB, angiotensin receptor blocker; BMI, body mass index; CABG, coronary artery bypass grafting; CAD, coronary artery disease; CKD, chronic kidney disease; CVA, cerebrovascular accident; Hb, hemoglobin; HbA1c, hemoglobin A1c; hs-CRP, high-sensitivity c-reactive protein; hs-troponin I, high-sensitivity troponin I; hs-troponin T, high-sensitivity troponin T; INR, international normalized ratio; IQR, interquartile range; LDL, low-density lipoprotein; LT, lysis time; MI, myocardial infarction; NSTEMI, non-ST segment elevation myocardial infarction; OT, occlusion time; PAD, peripheral arterial disease; PCI, percutaneous coronary intervention; PT, prothrombin time; SD, standard deviation; STEMI, ST-segment elevation myocardial infarction; WCC, white cell count.

Note: CKD defined as creatinine >177 μmol/L. Prior aspirin defined as regular use prehospitalization. Family history of premature CAD defined as a diagnosis of CAD in a first-degree relative <60 years. Values in bold are significant (i.e., *p* < 0.05).

**Table 3:** Baseline clinical and laboratory characteristics of patients with NSTEMI

	Western patients ( *n* = 95)	East Asian patients ( *n* = 100)	*p* -Value
*Clinical characteristics*			
Age, y (SD)	67.1 (12.9)	69.4 (11.0)	0.193
Male, *n* (%)	69 (72.6)	76 (76.0)	0.592
BMI, kg/m ^2^ (SD)	28.4 (5.9)	24.8 (3.5)	**<0.001**
Smoking, *n* (%)	19 (20.0)	18 (180)	0.724
Hypertension, *n* (%)	50 (52.6)	61 (61.0)	0.240
Diabetes, *n* (%)	27 (28.4)	38 (38.0)	0.158
Hypercholesterolemia, *n* (%)	25 (26.3)	19 (19.0)	0.224
Family history of premature CAD, *n* (%)	26 (27.4)	19 (19.0)	0.167
Angina, *n* (%)	11 (11.6)	17 (17.0)	0.283
Prior MI, *n* (%)	17 (17.9)	17 (17.0)	0.871
Prior PCI, *n* (%)	16 (16.8)	16 (16.0)	0.876
Prior CABG, *n* (%)	3 (3.2)	0 (0.0)	0.228
CKD, *n* (%)	4 (4.2)	5 (5.0)	0.796
PAD, *n* (%)	2 (2.1)	0 (0.0)	0.472
CVA, *n* (%)	3 (3.2)	6 (6.0)	0.347
*Laboratory characteristics*			
Hb, g/dL (IQR)	14.4 (13.4–15.2)	13.3 (12.0–14.6)	**<0.001**
WCC, ×10 ^9^ /L (IQR)	8.8 (7.3–11.2)	7.9 (6.1–9.2)	**0.002**
Platelets, ×10 ^9^ /L (IQR)	266 (207–303)	212 (183–244)	**<0.001**
INR (IQR)	1.0 (1.0–1.0)	1.0 (0.9–1.0)	**0.002**
PT, s (IQR)	10.9 (10.5–11.2)	13.0 (12.4–13.6)	**<0.001**
APTT, s (IQR)	23.4 (22.0–24.6)	35.3 (32.3–37.8)	**<0.001**
Fibrinogen, g/L (IQR)	3.4 (2.9–4.0)	3.5 (3.1–4.2)	0.510
Creatinine, μmol/L (IQR)	88 (71–102)	87 (71–104)	0.982
hs-CRP, mg/L (IQR)	3 (1–6.9)	4 (2.6–7.7)	**0.033**
HbA1c, mmol/mol (IQR)	39 (37–50)	44 (39–53)	0.336
Glucose, mmol/L (IQR)	6.3 (5.6–7.4)	6.8 (5.8–8.8)	0.133
Total cholesterol, mmol/L (IQR)	4.3 (3.1–5.3)	3.7 .3.3–4.7()	0.291
LDL cholesterol, mmol/L (IQR)	2.4 (1.4–3.4)	2.2 (1.7–3.0)	0.942
Troponin (peak)			
hs-troponin T, ng/L (IQR)	106 (46–340)	n/a	
hs-troponin I, ng/L (IQR)	n/a	(5,879 (1,162–15,990)	
*Thrombotic status*			
OT, s (IQR)	580 (474–712)	734 (541–866)	**<0.001**
LT, s (IQR)	1,898 (1,614–2,806)	1,519 (1,058–2,508)	**0.004**
*Admission medications*			
Aspirin, *n* (%)	28 (29.5)	73 (73.0)	**<0.001**
*Discharge medications*			
Aspirin, *n* (%)	92 (96.8)	93 (93.0)	0.375
Clopidogrel, *n* (%)	22 (23.2)	80 (80.0)	**<0.001**
Ticagrelor, *n* (%)	66 (69.5)	7 (7.0)	**<0.001**
Prasugrel, *n* (%)	0 (0.0)	13 (13.0)	**<0.001**
Beta-blocker, *n* (%)	79 (83.2)	65 (65.0)	**0.006**
ACEi/ARB, *n* (%)	80 (84.2)	66 (66.0)	**0.005**
Statin, *n* (%)	85 (895)	89 (89.0)	1.000

Abbreviations: ACEi, angiotensin-converting enzyme inhibitor; ACS, acute coronary syndrome; APTT, activated partial thromboplastin time; ARB, angiotensin receptor blocker; BMI, body mass index; CABG, coronary artery bypass grafting; CAD, coronary artery disease; CKD, chronic kidney disease; CVA, cerebrovascular accident; Hb, hemoglobin; HbA1c, hemoglobin A1c; hs-CRP, high sensitivity c-reactive protein; hs-troponin I, high sensitivity troponin I; hs-troponin T, high sensitivity troponin T; INR, international normalized ratio; IQR, interquartile range; LDL, low density lipoprotein; LT, lysis time; MI, myocardial infarction; NSTEMI, non-ST segment elevation myocardial infarction; OT, occlusion time; PAD, peripheral arterial disease; PCI, percutaneous coronary intervention; PT, prothrombin time; SD, standard deviation; STEMI, ST-segment elevation myocardial infarction; WCC, white cell count.

Note: CKD defined as creatinine >177 μmol/L. Prior aspirin defined as regular use prehospitalization. Family history of premature CAD defined as a diagnosis of CAD in a first-degree relative <60 years. Values in bold are significant (i.e., *p* < 0.05).

### Thrombotic Status

Although within the normal range, hemoglobin, white cell and platelet counts were lower, coagulation assays were more prolonged in East Asian patients, compared to Western patients ( Table 1 ). Fibrinogen levels were lower in East Asian patients than Westerners. In East Asian patients, OT was significantly longer than in Westerners, with no significant difference overall in LT between the two cohorts. Among STEMI patients, OT and LT were both much longer in East Asian patients than in Western patients. Among NSTEMI patients, OT was longer but LT shorter in East Asian compared to Western patients.

### Clinical Outcomes

Adverse cardiovascular events occurred significantly more frequently in Western than in East Asian patients, driven by a higher rate of cardiovascular death ( Table 4 ). Overall, the rate of bleeding complications was low, and similar in East Asian and Western patients. Due to few bleeding events, we could not assess a relationship between bleeding events and OT or LT.

**Table 4:** Clinical outcomes at 1 year in patients with myocardial infarction, based on ethnicity (statistically significant values shown in bold)

	All patients ( *n* = 515)	Westerners ( *n* = 255)	East Asians ( *n* = 260)	*p* -Value
MACE	21 (4.08%)	16 (6.27%)	5 (1.92%)	**0.014**
Cardiovascular death	10 (1.94%)	9 (3.53%)	1 (0.38%)	**0.010**
ACS	7 (1.36%)	5 (1.96%)	2 (0.77%)	0.281
TIA/CVA	4 (0.78%)	2 (0.78%)	2 (0.77%)	1.000
Further PCI	1 (0.19%)	1 (0.39%)	0 (0.0%)	0.495
Major bleeding (BARC Type 3–5)	2 (0.39%)	1 (0.39%)	1 (0.38%)	1.000
All-cause death	17 (3.30%)	14 (5.49%)	3 (1.15%)	**0.006**

Abbreviations: ACS, acute coronary syndrome; BARC, Bleeding Academic Research Consortium; CVA, cerebrovascular accident; ISR, in-stent restenosis; MACE, major adverse cardiovascular events; PCI, percutaneous coronary intervention; TIA, transient ischemic attack.

### Relationship between Thrombotic Status and Adverse Cardiovascular Outcomes

**Fig. 1:**
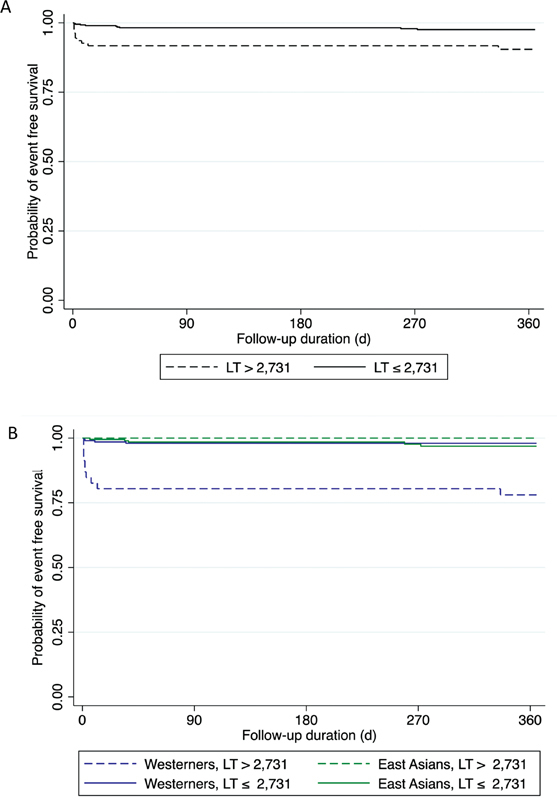
Kaplan–Meier curve showing relationship between endogenous fibrinolysis time (LT) and event-free survival in **(A)** all patients with ACS **(B)** in East Asian and Western patients with ACS. ACS, acute coronary syndrome.

Using ROC analysis, the optimal LT cut-point to predict MACE was 2,731 seconds (area under the ROC curve [AUC]: 0.67) ( Table 5 ). In the group as a whole, LT was predictive of MACE with HR 4.24 (95% CI: 1.72–10.43, *p* = 0.002) ( Fig. 1A ) driven by cardiovascular death (HR 15.28, 95% CI: 3.24- 71.96, *p* = 0.01) ( Table 6 ), and this remained a significant and independent risk after adjustment for univariate risk factors that were also independently associated with MACE (age, sex, race, diabetes, chronic kidney disease, prior angina, prior coronary artery bypass graft, and fibrinogen) (HR: 3.63, 95% CI: 1.17–11.31, *p* = 0.026) ( Supplementary Table S1 , available in the online version). Subgroup analysis by ethnicity showed that in Western patients, LT was strongly predictive of MACE (HR: 12.02, 95% CI: 3.77–38.34, *p* < 0.0001), even after adjustment for risk factors (HR: 10.25, 95% CI: 3.08–34.15, *p* < 0.001) but LT was not predictive of MACE in East Asian patients ( Table 7 , Fig. 1B ). Amongst Westerners, 18% had LT greater than 2,731 seconds, whilst 24% of the East Asian patients had LT above this level ( Table 7 ). The relationship between LT and cardiovascular outcomes in East Asian patients was very weak (AUC: 0.53), but this may have been driven by the very low MACE rate in this cohort ( Table 4 ). Subgroup analysis by AMI type showed that LT was a stronger predictor of MACE in STEMI patients than in NSTEMI patients, both in Westerners (AUC: 0.85 vs. 0.70) and in East Asian patients (AUC: 0.60 vs. 0.58) ( Table 5 ).

**Table 5:** Usefulness of thrombotic status (lysis time and occlusion time) for predicting major adverse cardiovascular events in different populations

	Optimal LT cut-off	Sensitivity	Specificity	Youden index (J)	C-index (AUC), [95% CI]	Optimal OT cut-off	Sensitivity	Specificity	Youden index (J)	C-index (AUC), [95% CI]
All patients	2,731	0.53	0.81	0.333	0.67, [0.55–0.78]	181	0.95	0.07	0.013	0.51, [0.46–0.56]
Westerners	2,730	0.71	0.86	0.579	0.79, [0.66–0.91]	181	0.93	0.09	0.014	0.51, [0.45–0.59]
East Asians	335	1.00	0.06	0.063	0.53, [0.51–0.54]	457	1.00	0.23	0.227	0.61, [0.59–0.64]
Westerners STEMI	2,686	0.75	0.95	0.696	0.85, [0.72–0.98]	181	0.92	0.13	0.045	0.52, [0.44–0.61]
East Asians STEMI	1,225	1.00	0.19	0.191	0.60, [0.57–0.63]	457	1.00	0.29	0.287	0.64, [0.60–0.68]
Westerners NSTEMI	1,816	1.00	0.41	0.409	0.70, [0.64–0.74]	616	1.00	0.59	0.586	0.79, [0.72–0.83]
East Asians NSTEMI	2,039	0.50	0.66	0.163	0.58, [0.09–1.00]	471	1.00	0.15	0.153	0.58, [0.54–0.61]

Abbreviations: AUC, area under the curve; CI, confidence intervals; LT, lysis time; NSTEMI, non-ST segment elevation myocardial infarction; OT, occlusion time; STEMI, ST-segment elevation myocardial infarction.

Note: Optimal cut-points with corresponding sensitivity, specificity, Youden index, and c-index values shown.

**Table 6:** Clinical outcomes at 1-year in patients with myocardial infarction in relation to endogenous fibrinolysis (statistically significant values shown in bold)

Adverse event	Whole group ( *n* = 515)	LT ≤ 2,731 ( *n* = 405)	LT > 2,731 ( *n* = 110)	HR	95% CI	*p* -Value
MACE	21 (4.08%)	10 (2.46%)	11 (10.00%)	4.24	1.72–10.43	**0.002**
Cardiovascular death	10 (1.94%)	2 (0.49%)	8 (7.27%)	15.28	3.24–71.96	**0.001**
ACS	7 (1.36%)	5 (1.23%)	2 (1.82%)	1.51	0.29–7.79	0.622
TIA/CVA	4 (0.78%)	3 (0.74%)	1 (0.91%)	1.29	0.13–12.39	0.826
Further PCI	1 (0.19%)	0 (0.00%)	1 (0.91%)	NA		
Major bleeding (BARC Type 3–5)	2 (0.39%)	1 (0.24%)	1 (0.91%)	3.91	0.24–62.47	0.335
All-cause death	17 (3.30%)	9 (2.22%)	8 (7.27%)	3.43	1.32–8.89	**0.011**

Abbreviations: ACS, acute coronary syndrome; BARC, Bleeding Academic Research Consortium; CI, confidence interval; CVA, cerebrovascular accident; HR, hazard ratio; ISR, in-stent restenosis; LT, lysis time; MACE, major adverse cardiovascular events; PCI, percutaneous coronary intervention; TIA, transient ischemic attack.

**Table 7:** Clinical outcomes at 1-year in East Asian and Western patients, in relation to endogenous fibrinolysis (statistically significant values shown in bold)

Adverse event	All patients ( *n* = 515)	East Asian ( *n* = 260)				Western ( *n* = 255)			
		All ( *n* = 260)	LT ≤ 2,731 ( *n* = 197)	LT > 2,731 ( *n* = 63)	*p* -Value	All ( *n* = 255)	LT ≤ 2,731 ( *n* = 208)	LT > 2,731 ( *n* = 47)	*p* -Value
MACE	21 (4.08%)	5 (1.92%)	5 (2.54%)	0 (0.00%)	0.340	16 (6.27%)	5 (2.40%)	11 (23.40%)	**0.000**
Cardiovascular death	10 (1.94%)	1 (0.38%)	1 (0.51%)	0 (0.00%)	1.000	9 (3.53%)	1 (0.48%)	8 (17.02%)	**0.000**
ACS	7 (1.36%)	2 (0.77%)	2 (1.02%)	0 (0.00%)	1.000	5 (1.96%)	3 (1.44%)	2 (4.26%)	0.230
TIA/CVA	4 (0.78%)	2 (0.77%)	2 (1.02%)	0 (0.00%)	1.000	2 (0.78%)	1 (0.48%)	1 (2.13%)	0.335
Further PCI	1 (0.19%)	0 (0.0%)	0 (0.00%)	0 (0.00%)	NA	1 (0.39%)	0 (0.00%)	1 (2.13%)	0.184
Major bleeding (BARC Type 3 -5)	2 (0.39%)	1 (0.38%)	0 (0.00%)	1 (1.59%)	0.242	1 (0.39%)	1 (0.48%)	0 (0.00%)	1.000
All-cause death	17 (3.30%)	3 (1.15%)	3 (1.52%)	0 (0.00%)	1.000	14 (5.49%)	6 (2.88%)	8 (17.02%)	**0.001**

Abbreviations: ACS, acute coronary syndrome; BARC, Bleeding Academic Research Consortium; CVA, cerebrovascular accident; ISR, in-stent restenosis; LT, lysis time; MACE, major adverse cardiovascular events; PCI, percutaneous coronary intervention; TIA, transient ischemic attack.

In the group as a whole, OT was not related to MACE or its components (AUC: 0.51) ( Tables 5 and 8 ), although OT was more closely related to MACE in East Asian than in Western patients (AUC: 0.61 vs. 0.51). Although there were baseline differences in aspirin use prior to admission between the East Asian and Western cohorts, further analysis excluding those patients who were taking aspirin prior to admission showed that OT remained unrelated to MACE ( Supplementary Tables S2 and S3, available in the online version ). In East Asian patients, OT was more closely related to adverse events in STEMI than NSTEMI (AUC: 0.64 vs. 0.58) and in Westerners in NSTEMI than in STEMI patients (AUC: 0.79 vs. 0.52) ( Table 5 ). In neither Western nor in East Asian patients, could OT differentiate between patients with and without MACE over the follow-up period ( Table 9 ). Furthermore, there was no difference in OT at discharge by type of P2Y _12_ inhibitor used (clopidogrel 570 [422–791] seconds vs. ticagrelor 479 [357–659] seconds vs. prasugrel 543 [434–815] seconds). The relationship between cardiovascular events according to different P2Y _12_ inhibitors upon discharge is shown in Supplementary Table S4 (available in the online version).

**Table 8:** Relationship between adverse events at 1-year and occlusion time (statistically significant values shown in bold)

Adverse event	All patients ( *n* = 515)	OT ≤ 181 ( *n* = 39)	OT > 181 ( *n* = 476)	HR	95% CI	*p* -Value
MACE	21 (4.08%)	1 (2.56%)	20 (4.20%)	1.50	0.20–11.23	0.694
Cardiovascular death	10 (1.94%)	1 (2.56%)	9 (1.89%)	0.75	0.09–5.89	0.781
ACS	7 (1.36%)	0	7 (1.47%)	NA	NA	NA
TIA/CVA	4 (0.78%)	0	4 (0.84%)	NA	NA	NA
Further PCI	1 (0.19%)	0	1 (0.21%)	NA	NA	NA
Major bleeding (BARC 3-5)	2 (0.39%)	0	2 (0.42%)	NA	NA	NA
All-cause death	17 (3.30%)	3 (7.69%)	14 (2.94%)	0.39	0.11–1.37	0.141

Abbreviations: ACS, acute coronary syndrome; BARC, Bleeding Academic Research Consortium; CI, confidence interval; CVA, cerebrovascular accident; HR, hazard ratio; ISR, in-stent restenosis; MACE, major adverse cardiovascular events; NA, not applicable; OT, occlusion time; PCI, percutaneous coronary intervention; TIA, transient ischemic attack.

**Table 9:** Relationship between adverse cardiovascular events at 1-year follow-up and optimal OT cut-point, by ethnicity

Adverse event	All patients ( *n* = 515)	East Asian ( *n* = 260)				Western ( *n* = 255)			
		All ( *n* = 260)	OT ≤ 457 ( *n* = 57)	OT > 457 ( *n* = 203)	*p* -Value	All ( *n* = 255)	OT ≤ 181 ( *n* = 27)	OT > 181 ( *n* = 228)	*p* -Value
MACE	21 (4.08%)	5 (1.92%)	0 (0.00%)	5 (2.46%)	0.589	16 (6.27%)	1 (3.70%)	15 (6.58%)	1.000
Cardiovascular death	10 (1.94%)	1 (0.38%)	0 (0.00%)	1 (0.49%)	1.000	9 (3.53%)	1 (3.70%)	8 (3.51%)	1.000
ACS	7 (1.36%)	2 (0.77%)	0 (0.00%)	2 (0.99%)	1.000	5 (1.96%)	0 (0.00%)	5 (2.19%)	1.000
TIA/CVA	4 (0.78%)	2 (0.77%)	0 (0.00%)	2 (0.99%)	1.000	2 (0.78%)	0 (0.00%)	2 (0.88%)	1.000
Further PCI	1 (0.19%)	0 (0.0%)	0 (0.00%)	0 (0.00%)	NA	1 (0.39%)	0 (0.00%)	1 (0.44%)	1.000
Major bleeding (BARC Type 3 -5)	2 (0.39%)	1 (0.38%)	0 (0.00%)	1 (0.49%)	1.000	1 (0.39%)	0 (0.00%)	1 (0.44%)	1.000
All-cause death	17 (3.30%)	3 (1.15%)	0 (0.00%)	3 (1.48%)	1.000	14 (5.49%)	3 (11.11%)	11 (4.82%)	0.174

Abbreviations: ACS, acute coronary syndrome; BARC, Bleeding Academic Research Consortium; CVA, cerebrovascular accident; ISR, in-stent restenosis; MACE, major adverse cardiovascular events; NA, not applicable; OT, occlusion time; PCI, percutaneous coronary intervention; TIA, transient ischemic attack.

### Relationship of Lysis Time to Clinical and Laboratory Characteristics

When relating LT to the clinical and laboratory characteristics shown in Table 1 , LT was related to acute coronary syndrome (ACS) presentation (STEMI, *r* = 0.11, *p* = 0.018), and weakly correlated with hs-CRP, total cholesterol, and peak hs-troponin T in Western patients but not with other hematological or biochemical parameters on admission ( Table 10 ). Subgroup analysis of the relationship between LT and clinical and laboratory characteristics in STEMI and NSTEMI patients is shown in Supplementary Tables S5 and S6, available in the online version . Subgroup analysis by ethnicity revealed that in Western patients, LT was weakly related to ACS presentation, BMI, diabetes, creatinine, hs-CRP, troponin, and coagulation assays ( Supplementary Table S7 , available in the online version). In contrast, in East Asian patients, LT was weakly related to ACS (STEMI) presentation ( *r* = 0.129, *p* = 0.038), blood glucose ( *r* = 0.139, *p* = 0.032), and activated partial thromboplastin time ( *r* = 0.128, *p* = 0.04) on presentation.

**Table 10:** Correlation of lysis time with clinical and laboratory characteristics in the entire cohort (statistically significant values shown in bold)

	LT (r)	*p* -Value
*Baseline characteristics*		
Age	0.0243	0.5842
Male	0.0290	0.5142
Race	0.0860	0.0522
BMI	0.0775	0.0838
Smoking	0.0327	0.4619
Hypertension	0.0052	0.9066
Diabetes	0.0671	0.1305
Hypercholesterolemia	−0.0037	0.9338
Family history of premature CAD	−0.0314	0.4786
Angina	0.0391	0.3778
Prior MI	0.0337	0.4472
Prior PCI	0.0311	0.4836
Prior CABG	0.0437	0.3245
CKD	0.0616	0.1645
PAD	0.0258	0.5603
CVA	0.0293	0.5087
ACS presentation	**0.1051**	**0.0176**
Aspirin (on admission)	−0.0056	0.9000
*Laboratory characteristics*		
Peak hs-Troponin T	**0.1457**	**0.0272**
Peak hs-Troponin I	0.0735	0.2377
Hs-CRP	**0.1433**	**0.0013**
Creatinine	0.0649	0.1433
HbA1c	0.0943	0.2388
Glucose	0.0830	0.1574
Platelets	−0.0513	0.2477
INR	0.0433	0.3479
PT	0.0185	0.7014
APTT	0.0192	0.6781
Fibrinogen	−0.0134	0.7731
Hb	0.0053	0.9055
WCC	−0.0178	0.6892
Total cholesterol	− **0.1167**	**0.0147**
LDL cholesterol	−0.0904	0.1176
*Thrombotic status*		
OT	0.0010	0.9830

Abbreviations: ACEi, angiotensin-converting enzyme inhibitor; ACS, acute coronary syndrome; APTT, activated partial thromboplastin time; ARB, angiotensin receptor blocker; BMI, body mass index; CABG, coronary artery bypass grafting; CAD, coronary artery disease; CKD, chronic kidney disease; CVA, cerebrovascular accident; Hb, hemoglobin; HbA1c, hemoglobin A1c; hs-CRP, high-sensitivity c-reactive protein; hs-troponin I, high-sensitivity troponin I; hs-troponin T, high-sensitivity troponin T; INR, international normalized ratio; IQR, interquartile range; LDL, low-density lipoprotein; MI, myocardial infarction; NSTEMI, non-ST segment elevation myocardial infarction; OT, occlusion time; PAD, peripheral arterial disease; PCI, percutaneous coronary intervention; PT, prothrombin time; SD, standard deviation; STEMI, ST-segment elevation myocardial infarction; WCC, white cell count.

Note: CKD defined as creatinine >177 μmol/L. Family history of premature CAD defined as a diagnosis of CAD in a first-degree relative <60 years. Values in bold are significant (i.e., *p* < 0.05).

## Discussion

Among patients with MI, the rate of adverse events over a 12-month follow-up was much lower in East Asian patients than in Westerners, and this is accompanied by marked differences in global thrombotic profile. In this, the first study comparing global thrombotic profile in East Asian and Western patients with AMI, we show that East Asian patients exhibit reduced thrombotic occlusion, evidenced by significantly longer OT and generally similar endogenous fibrinolysis to Westerners.

Prolonged endogenous fibrinolysis was an independent predictor of adverse cardiovascular events, with a fourfold increased risk, driven predominantly by cardiovascular death. In both Western and East Asian cohorts, the predictive value of LT was greater in STEMI than in NSTEMI patients. Although endogenous fibrinolysis time was similar in the two cohorts, it was a much stronger predictor of future adverse cardiovascular events in Western than in East Asian patients. This could be attributable to the very few adverse events in East Asian patients compared to Westerners. A previous study in patients with stable coronary disease showed that viscoelastic properties of whole blood, measured by thromboelastography, differed between East Asian and Caucasian patients, with delayed initiation of clot formation, lower clot strength, and faster clot lysis in East Asian patients. 33 High platelet-fibrin clot strength was a significant predictor of ischemic events over a 3-year follow-up, with high clot strength prevalence being 50% lower in East Asians than in Caucasians.

Surprisingly, hs-CRP was higher in East Asian than in Western patients at baseline. Endogenous fibrinolysis was weakly correlated with hs-CRP in both STEMI and NSTEMI patients, but only in Western and not East Asian patients. This finding is supported by previous studies in Caucasian patients that have shown evidence of bi-directional cross-talk between coagulatory and inflammatory pathways, which could help guide pharmacological strategies to treat hypofibrinolysis in these patients. 27 34

East Asian patients showed prolonged OT in both STEMI and NSTEMI, compared to Western patients, and despite less potent antiplatelet medications. Although LT was overall similar in Western and East Asian patients, the relatively high OT in East Asians may offer cardiovascular protection. More potent P2Y _12_ inhibitors (prasugrel or ticagrelor) were taken by 37.3% Korean patients compared to 73.7% of Western patients on discharge. However, OT was overall not related to adverse cardiovascular events. Previously, we have shown prolongation of OT in Western patients from the time of initial admission with STEMI to the time of discharge, which almost certainly reflects the effects of DAPT treatment, initiated during admission. 27 Furthermore, in earlier work, we have shown increasing prolongation of OT with more potent oral P2Y _12_ inhibitors, with no significant effect of P2Y _12_ inhibitors on LT. 35 The fact that OT on presentation was not predictive of adverse cardiovascular events during follow-up is likely explained by the further modulation of OT by DAPT during the hospitalization and beyond, such that the baseline OT is no longer reflective of risk. It is therefore also surprising that Korean patients, amongst whom fewer were taking more potent P2Y _12_ inhibitors, demonstrated a longer OT than Western patients, indicating a genetic basis. In earlier work, comparing thrombotic profiles in healthy volunteers, OT was longer in Japanese than in Western patients. 36 Whilst absolute OT values between the results of that study from >10 years ago and the study now in ACS patients cannot be directly compared, the combined results indicate that both in healthy volunteers and in ACS patients, OT is longer in East Asians than in westerners. Furthermore, this prolonged OT in East Asian individuals is seen both in the absence of antiplatelet medication (healthy volunteers) and in STEMI patients, who were sampled before the onset of DAPT effect. This suggests an ethnic difference in OT in East Asian and Western individuals, that is unaffected by disease process. On the other hand, whilst that earlier paper showed that LT was longer in East Asian than in Western healthy individuals, we found that in the setting of ACS, especially in STEMI patients, it was longer in Western than East Asian patients. In essence, the longer LT in Western patients with ACS, compared to healthy Western volunteers, associated with the higher rate of MI and the higher rate of recurrent adverse events post-MI supports the concept that increased LT in Westerners is implicated in the pathogenesis of cardiovascular thrombotic events.

The prolonged OT seen here in Korean patients may therefore represent a genetic profile which antiplatelet medication further accentuates. Amongst patients with atrial fibrillation taking oral anticoagulation, OT was significantly prolonged in Japanese patients compared to white Europeans. 37 We therefore postulate that the baseline prolonged OT seen in East Asian patients may afford cardiovascular protection, although because of the very low event rate in this population, we did not show a significant predictive value of OT for MACE. Clearly, future larger studies would be needed to definitively address this.

Larger cohort studies in East Asian patients with STEMI, with longer duration of follow-up, adequately powered to assess the prognostic significance of thrombotic status on cardiovascular outcomes are needed. Furthermore, large studies are also needed in East Asian patients to assess the relationship between thrombotic status and the risk of bleeding events, which this study was underpowered to evaluate.

### Limitations

There are notable limitations to our study. This was a two-center collaboration with a relatively small sample size and the low rate of adverse events over the follow-up period, particularly in Korean patients, limits the conclusions we can draw about the usefulness of thrombotic status results in predicting cardiovascular outcomes in these populations. The populations were generally well matched, but with significantly higher BMI in Western patients than in East Asians, as well as higher prevalence of hyperlipidemia and a positive family history of cardiovascular disease. These are recognized inter-ethnic differences and whilst they may be a confounder, these features are typical characteristics of the populations studied. Hemoglobin and platelet count were both lower with coagulation tests slightly longer in Korean patients than in Western patients. Whilst still within the normal range, this could have influenced outcomes, although there was no relationship between any of these markers and OT or LT. Medications on discharge were different in the two cohorts, with more potent P2Y _12_ inhibitors given to Westerners. Although we would have expected this to skew the results in favor of fewer events in Western patients, and the opposite was observed, and so it is unlikely to have confounded the results. Although follow-up was 100% complete in both cohorts for MACE, as reported by patients or as evidenced by case notes, events that may not have been reported by patients and which were not documented in local case records may have been missed.

## Conclusion

In the first study to assess inter-ethnic differences in global thrombotic status in patients with AMI, we show that East Asian (Korean) patients exhibit a different thrombotic profile to white Caucasian western patients, and this is associated with a lower rate of recurrent cardiovascular events.

Future large studies will be required to identify the cardiovascular risk associated with relatively different global thrombotic profiles in East Asian and white Caucasian populations. This may allow differential tailoring of antithrombotic medications, by ethnicity, to reduce ischemic and bleeding events.

## Perspectives

### Competency in Medical Knowledge

East Asians have a lower risk of ischemic heart disease and a higher risk of bleeding with antithrombotic medications, compared to Westerners. The underlying mechanisms behind these ethnic differences are incompletely understood. We show that East Asian patients exhibit a markedly different thrombotic profile in blood compared to Westerners, which may explain their lower rate of cardiovascular events.

### Translational Outlook

Future large studies are required to identify the cardiovascular risk associated with relatively different global thrombotic profiles in East Asian and Western populations. This may allow differential tailoring of antithrombotic medications, by ethnicity, to reduce ischemic and bleeding events.

## Notes

**What is known on this topic?**

- East Asians have a lower risk of ischemic heart disease and a higher risk of bleeding with antithrombotic medications, compared to Westerners.
- The underlying mechanisms behind these ethnic differences, and how they contribute to cardiovascular outcomes, are incompletely understood.

**What does this paper add?**

- We show that East Asian patients exhibit a markedly different thrombotic profile in blood compared to Westerners, which may explain their lower rate of cardiovascular events after a heart attack.
- Future large studies are required to identify the cardiovascular risk associated with relatively different global thrombotic profiles in East Asian and Western populations, which may allow tailoring of antithrombotic medications, by ethnicity, to reduce ischemic and bleeding events.
