# Supplementary material for: Ethnic Differences in Thrombotic Profiles of Acute Coronary Syndrome Patients and Relationship to Cardiovascular Outcomes: A Comparison of East Asian and White subjects
Source: Thromb Haemost. 2023 Dec 29;124(6):501–16. doi: 10.1055/s-0043-1777794 (PMC11126334; doi:10.1055/s-0043-1777794)
Supplement: Supplementary file 1 — Supplementary Material [file 10-1055-s-0043-1777794-s23050213.pdf]

Supplemental Material

Supplementary Definition of Components of the Primary Endpoint

Cardiovascular death was defined as death in the presence of acute coronary syndrome, significant arrhythmia, or refractory congestive heart failure, or death attributed to cardiovascular cause at postmortem.

New myocardial infraction or re-infarction was defined according to the universal definition as the detection of rise and/or fall of troponin T with at least one value >99th percentile of the upper reference limit and with at least one of the following: symptoms of ischemia, new or presumed new significant ST-T changes or new left bundle branch block, development of pathological Q-waves, imaging evidence of new loss of viable myocardium, or new regional wall motion abnormality, identification of intracoronary thrombus at angiography, or stent thrombosis associated with myocardial ischemia detected by angiography.[32] Specifically re-infarction following the index pPCI during the same hospitalization (myocardial infarction type 4) was defined as recurrence of symptoms of ischemia and/or new or presumed new electrocardiogram (ECG) changes as defined above distinct from the ECG changes secondary to the index event, together with either (1) an increase in troponin greater than 3×99th percentile upper reference limit and re-elevation by at least 20% from previous baseline following a decrease from the peak value (myocardial infarction type 4a) or (2) angiographic evidence of stent thrombosis as shown by new thrombus, vessel occlusion, or sub-total occlusion (myocardial infarction type 4b).

Stroke was defined as an acute focal brain infarction with one of the following: sudden onset of new focal neurologic deficit, with clinical or imaging evidence of infarction lasting $\geq$24 hours and not attributable to a nonischemic cause, or new focal neurologic deficit lasting <24 hours and not attributable to a nonischemic cause but accompanied by neuroimaging evidence of new brain infarction.

Supplementary Table S1 Univariate predictors of major adverse cardiovascular events (MACE) (only statistically significant results shown)

	Hazard ratio (95% confidence interval)	*p*-Value
Clinical characteristics		
Age	1.06 (1.02–1.10)	0.005
Sex	3.57 (1.45–8.78)	0.006
Race	0.35 (0.13–0.97)	0.043
Diabetes, *n* (%)	2.30 (0.92–5.72)	0.073
Hypercholesterolemia	2.45 (0.99–6.02)	0.051
Angina	2.97 (1.07–8.24)	0.037
Prior CABG	9.06 (1.21–68.05)	0.032
CKD	10.36 (3.72–28.82)	0.000
Fibrinogen	1.44 (1.06–1.96)	0.021

Abbreviations: CABG, coronary artery bypass grafting; CAD, coronary artery disease; CKD, chronic kidney disease.
Note: CKD defined as creatinine >177 μmol/L.

Supplementary Table S2 Relationship between adverse events at 1 year and occlusion time, in subgroup of patients not taking aspirin on admission

Adverse event	All patients ($n = 343$)	OT $\leq$ 181 ($n = 25$)	OT > 181 ($n = 318$)	HR	95% CI	*p*-Value
MACE	12	0	12 (3.77%)	NA	NA	NA
Cardiovascular death	4 (1.17%)	0	4 (1.26%)	NA	NA	NA
ACS	6 (1.75%)	0	6 (1.89%)	NA	NA	NA
TIA/CVA	2 (0.58%)	0	2 (0.63%)	NA	NA	NA
Further PCI	0	0	0	NA	NA	NA
Major bleeding (BARC 3-5)	2 (0.58%)	0	2 (0.63%)	NA	NA	NA
All-cause death	9 (2.62%)	1 (4.00%)	8 (2.52%)	0.65	0.08–5.19	0.68

Abbreviations: ACS, acute coronary syndrome; BARC, Bleeding Academic Research Consortium; CI, confidence interval; CVA, cerebrovascular accident; HR, hazard ratio; ISR, in-stent restenosis; MACE, major adverse cardiovascular events; NA, not applicable; OT, occlusion time; PCI, percutaneous coronary intervention; TIA, transient ischemic attack.

 © 2023. The Author(s).

Supplementary Table S3 Relationship between adverse cardiovascular events at 1-year follow-up and optimal OT cut-point, by ethnicity, in subgroup of patients not taking aspirin on admission

Adverse event	All patients ($n=343$)	East Asian ($n=146$)				Western ($n=197$)			
		All ($n=146$)	OT $\leq$ 457 ($n=40$)	OT > 457 ($n=106$)	p-Value	All ($n=197$)	OT $\leq$ 181 ($n=18$)	OT > 181 ($n=179$)	p-Value
MACE	12	2 (1.37%)	0 (0.00%)	2 (1.89%)	1.000	10 (5.08%)	0 (0.00%)	10 (5.59%)	1.000
Cardiovascular death	4 (1.17%)	0 (0.00%)	0 (0.00%)	0 (0.00%)	NA	4 (2.03%)	0 (0.00%)	4 (2.23%)	1.000
ACS	6 (1.75%)	1 (0.68%)	0 (0.00%)	1 (0.94%)	1.000	5 (2.54%)	0 (0.00%)	5 (2.79%)	1.000
TIA/CVA	2 (0.58%)	1 (0.68%)	0 (0.00%)	1 (0.94%)	1.000	1 (0.51%)	0 (0.00%)	1 (0.56%)	1.000
Further PCI	0 (0.00%)	0 (0.0%)	0 (0.00%)	0 (0.00%)	NA	0 (0.00%)	0 (0.00%)	0 (0.00%)	NA
Major bleeding (BARC Type 3–5)	2 (0.58%)	1 (0.68%)	0 (0.00%)	1 (0.94%)	1.000	1 (0.51%)	0 (0.00%)	1 (0.56%)	1.000
All-cause death	9 (2.62%)	1 (0.68%)	0 (0.00%)	1 (0.94%)	1.000	8 (4.06%)	1 (5.56%%)	7 (3.91%)	0.542

Abbreviations: ACS, acute coronary syndrome; BARC, Bleeding Academic Research Consortium; CVA, cerebrovascular accident; MACE, major adverse cardiovascular events; N/A, not applicable; PCI, percutaneous coronary intervention; OT, occlusion time, TIA, transient ischemic attack.

 © 2023. The Author(s).

Supplementary Table S4 Association between adverse cardiovascular events at 1-year follow-up according to different $P2Y_{12}$ inhibitors upon discharge (statistically significant values shown in bold)

	Western	East Asian	*p*-Value
Discharged on clopidogrel	*n* = 47	*n* = 158	
MACE	2 (4.26%)	2 (1.27%)	0.226
Cardiovascular death	1 (2.13%)	0	0.229
ACS	0	1 (0.63%)	1.000
TIA/CVA	1 (2.13%)	1 (0.63%)	0.407
Further PCI	0	0	n/a
Major bleeding (BARC Type 3–5)	1 (2.13%)	1 (0.63%)	0.407
All-cause death	5 (10.64%)	2 (1.27%)	**0.008**
Discharged on ticagrelor	*n* = 188	*n* = 51	
MACE	4 (2.13%)	2 (3.92%)	0.611
Cardiovascular death	0	0	n/a
ACS	4 (2.13%)	1 (1.96%)	1.000
TIA/CVA	0	1 (1.96%)	0.213
Further PCI	0	0	n/a
Major bleeding (BARC Type 3–5)	0	0	n/a
All-cause death	1 (0.53%)	0	1.000
Discharged on prasugrel	*n* = 0	*n* = 46	
MACE	0	1 (2.17%)	n/a
Cardiovascular death	0	1 (2.17%)	n/a
ACS	0	0	n/a
TIA/CVA	0	0	n/a
Further PCI	0	0	n/a
Major bleeding (BARC Type 3-5)	0	0	n/a
All-cause death	0	1 (2.17%)	n/a

Abbreviations: ACS, acute coronary syndrome; BARC, Bleeding Academic Research Consortium; CVA, cerebrovascular accident; MACE, major adverse cardiovascular events; N/A, not applicable; PCI, percutaneous coronary intervention; TIA, transient ischemic attack.

Supplementary Table S5 Correlation between LT and clinical and laboratory characteristics in patients with STEMI (Spearman pairwise correlation, only statistically significant results shown)

	LT (r)	*p*-Value
Baseline characteristics		
Race	0.2875	0.0000
Diabetes	0.1247	0.0256
Laboratory characteristics		
Hs-CRP	0.1294	0.0216
HbA1c	0.2417	0.0354
PT	0.1488	0.0113
aPTT	0.1769	0.0023

Abbreviations: aPTT, activated partial thromboplastin time; HbA1c, hemoglobin A1c; hs-CRP, high-sensitivity C-reactive protein; PT, prothrombin time.

Supplementary Table S6 Correlation between LT and clinical and laboratory characteristics in patients with NSTEMI (Spearman pairwise correlation, only statistically significant results shown)

	LT (r)	*p*-Value
Baseline characteristics		
Age	0.1428	0.0493
Race	−0.2123	0.0033
BMI	0.1658	0.0266
Laboratory characteristics		
Peak hs-troponin T (Western patients only)	0.2864	0.0065
Hs-CRP	0.1769	0.0154
INR	0.1855	0.0137

Abbreviations: BMI, body mass index; hs-CRP, high sensitivity C-reactive protein; hs-troponin T, high-sensitivity troponin T; INR, international normalized ratio; OT, occlusion time.

 © 2023. The Author(s).

Supplementary Table S7 Correlation between LT and clinical and laboratory characteristics in Western patients (Spearman pairwise correlation, only statistically significant results shown)

	LT (r)	*p*-Value
Baseline characteristics		
BMI	0.1541	0.0171
Diabetes	0.1427	0.0240
Angina	0.1553	0.0140
CKD	0.1242	0.0499
ACS presentation	0.3968	0.0000
Laboratory characteristics		
Peak hs-troponin T	−0.1457	0.0272
Hs-CRP	0.1535	0.0168
Creatinine	0.1407	0.0261
PT	−0.1916	0.0049
aPTT	−0.3281	0.0000

Abbreviations: ACS, acute coronary syndrome; aPTT, activated partial thromboplastin time; BMI, body mass index; CKD, chronic kidney disease; hs-CRP, high-sensitivity C-reactive protein; hs-troponin T, high-sensitivity troponin T; PT, prothrombin time.
Note: CKD defined as creatinine >177 µmol/L.

 © 2023. The Author(s).
